# 508 A Case Report of Trichosporon asahii Burn Infection: A Not-So-Fungi

**DOI:** 10.1093/jbcr/irac012.139

**Published:** 2022-03-23

**Authors:** Allison N Boyd, Todd A Walroth, Abbie Blunier, William H Harris, Brett C Hartman

**Affiliations:** Eskenazi Health, Indianapolis, Indiana; Eskenazi Health, Indianapolis, Indiana; Purdue University College of Pharmacy, West Lafayette, Indiana; Purdue University College of Pharmacy, West Lafayette, Indiana; Richard M. Fairbanks Burn Center, Indianapolis, Indiana

## Abstract

**Introduction:**

*Trichosporon asahii* is a yeast-like basidiomycete that can cause infections in immunocompromised hosts including superficial infections, pneumonia, meningitis, fungemia, and disseminated trichosporonosis. These infections have become more common over the last 30 years in patients with burns or other immunosuppressive conditions. Disseminated *T. asahii* is intrinsically difficult to treat and carries a mortality rate as high as 70% despite the availability of effective medications.

**Methods:**

A 61-year-old male was admitted after falling into a vat of pig manure which was 160 degrees F, resulting in approximately 75% total body surface area injury to his anterior and posterior trunk, and bilateral upper and lower extremities. Throughout his hospital course, he underwent surgical debridement on multiple occasions with placement of homograft, autograft, and spray keratinocytes. The earliest cultures to demonstrate *T. asahii* growth were obtained on post-burn day (PBD) 23 and growth continued until PBD 32. Quantitative colony counts ranged from 600-365000000 CFU/g from left lower and upper extremities and chest. Cultures were obtained in preparation for cultured epithelial autograft placement which occurred on PBD 36. Susceptibility testing was sent and the minimum inhibitory concentration (MIC) results are reported in Table 1. Clinical and Laboratory Standards Institute (CLSI) guidelines for interpretation of susceptibility are not available for *T. asahii*, but suggested breakpoints are summarized in Table 1.

**Results:**

On PBD 23, topical amphotericin B irrigation and voriconazole oral suspension were initiated. A voriconazole level was obtained on PBD 31, which was subtherapeutic, and dosing was subsequently adjusted. In total, the patient received 13 days of topical amphotericin B and 21 days of voriconazole for treatment. Subsequent cultures demonstrated microbiologic cure of *T. asahii*.

**Conclusions:**

This immunocompromised burn patient was successfully treated for *Trichosporon asahii* using a combination of topical and systemic antifungal agents.

